# Exploring preconception health beliefs amongst adults of childbearing age in the UK: a qualitative analysis

**DOI:** 10.1186/s12884-020-2733-5

**Published:** 2020-01-16

**Authors:** Laura McGowan, Emer Lennon-Caughey, Cheryl Chun, Michelle C. McKinley, Jayne V. Woodside

**Affiliations:** 1 Centre for Public Health, School of Medicine, Dentistry and Biomedical Science, Queen’s University Belfast, Belfast, BT12 6BJ UK; 2 Institute for Global Food Security, School of Biological Sciences, Queen’s University Belfast, Belfast, BT9 5BN UK; 3 Lecturer in Nutrition and Behaviour Change, Centre for Public Health (Institute for Global Food Security), Institute of Clinical Sciences, Queen’s University Belfast, Block A, 1st Floor, Room 01.027, Royal Victoria Hospital Site, Belfast, BT12 6BA UK; 4 National University of Ireland, Galway, H91 TK33 Ireland

**Keywords:** Preconception health and care, Pre-pregnancy health, Beliefs, Attitudes, Health behaviour, Nutrition, Males, Females, Lifestyle, Physical activity

## Abstract

**Background:**

‘Preconception health’ or ‘pre-pregnancy health’ are terms used to describe the health status of males and females prior to pregnancy. The goal of preconception health strategies is to optimise the health of future offspring via improved parental health, which may result from planned/unplanned pregnancies. Greater emphasis is being placed upon preconception health amongst research and public health, yet there is limited evidence on this topic from the perspective of UK adults. This research explored beliefs, knowledge and attitudes on preconception health amongst adults of childbearing age, drawn from the UK.

**Methods:**

A descriptive qualitative focus group study was undertaken with healthy males and females of childbearing age (18–45 years) between October 2018 and July 2019. Two groups were held in a rural location (one focus group, one mini focus group) and three groups held in an urban location (two focus groups, one mini focus group), with a range of males and females, with and without children. A semi-structured topic guide was devised based on previous literature. All groups were conducted with two researchers trained in qualitative research methods. Focus groups explored understanding/prior knowledge of preconception health, beliefs and attitudes towards preconception healthcare support and personal health. Focus groups were transcribed verbatim and analysed using thematic analysis.

**Results:**

Twenty-one males and females of childbearing age (aged 18 to 45 years) participated in the research. Discussions revealed a lack of comprehensive awareness of the importance of preconception health and a sense of reluctance to visit a doctor regarding the issue, favouring the internet, unless having problems conceiving. Five themes identified included: preconception education, preconception awareness, wider knowledge networks/support, optimal parental health, and attitudes/emotions towards preconception health. The roles of males regarding positive preconception care was not well understood.

**Conclusions:**

This study highlighted a lack of detailed awareness surrounding the importance of preconception health per se, despite general agreement that health status should be optimal at this time. It identified a willingness to learn more about preconception health, creating an opportunity to improve preconception healthcare awareness via evidence-based education, social media campaigns, and within healthcare systems in a life course approach.

## Background

Preconception or ‘pre-pregnancy’ health refers to the health of males and females at any point in time prior to a potential pregnancy [1]. Ultimately, the goal of preconception care (PCC) is to improve pregnancy outcomes and health in general through prevention of disease and management of risk factors that affect pregnancy outcome and the health of future offspring, resulting from both planned and unplanned pregnancies [2, 3]. These preventative PCC services are designed to reduce risk factors before pregnancy to increase the likelihood of successful conception, pregnancy outcomes and the prevention of developing chronic diseases for both the child and the mother during or following pregnancy [4].

Despite adults nowadays being deemed as more *‘health conscious’* [5] PCC is not given due prominence by health professionals nor the public [6]. Developments in obstetric healthcare have been notable, yet the rate of perinatal mortality in the UK has not changed significantly over recent years [7]. This has led researchers to suggest that babies’ health and insufficient promotion and knowledge of preconception health among adults of childbearing age are correlated [8].

In the United Kingdom (UK), more than six out of ten adults are classified as having overweight or obesity [9, 10]. This is found in many countries, where up to 50% of women are considered to have overweight or obesity when they become pregnant [11]. Obesity and poor nutrition greatly increase the risk of most major adverse maternal and perinatal outcomes such as the inability to conceive, pre-eclampsia and gestational diabetes, as well as difficulty during delivery, congenital abnormalities, stillbirth, low birth-weight, difficulty breastfeeding and maternal death [11, 12]. Furthermore, the health of the male prior to conception is becoming increasingly recognized as a factor for improving reproductive health, pregnancy and neo-natal outcomes [11, 13, 14]. Male fertility is closely linked to key aspects such as nutrition and body composition; an elevated body mass index (BMI) is associated with reduced sperm motility, increased sperm abnormality, increased sperm reactive oxygen species levels, reduced serum testosterone and increased estradiol concentrations [14].

Currently, PCC varies within and between different countries and health services. In many European countries, preconception policies, guidelines, services and recommendations are aimed towards women with existing chronic medical conditions [15]. In the UK, it is estimated that 62.4% of pregnancies amongst females aged 20–34 years were reported unplanned [16]. These figures highlight the need to create a culture of PCC to maximize chances of a healthy pregnancy. Recently, experts in the UK have called for the implementation of PCC strategies to positively impact preconception health [11, 17] such as supplementation and fortification of key vitamins/minerals in the diet and behavior change interventions [17]. It is suggested that these PCC strategies should be incorporated into a life course framework, involving individuals and motivating them to be more aware of preconception health at different life stages [17]. Yet, relatively little is known about public views on PCC strategies/behaviors (or preconception health more broadly), particularly in a UK context. Previous research from other countries, for example, Italy, has indicated that there is a significant lack of awareness about ensuring the body (of males and females) is in optimum condition for a potential pregnancy [18]. Furthermore, women tended to only worry about their own health and the health of the baby once a pregnancy has been discovered [18]. Research focusing on Australian males also highlighted limited knowledge on PCC behaviors and identified a severe lack of preconception healthcare promotion [19].

Given the significant gap regarding public views on preconception health and PCC in the UK, from a male and female perspective, this study aimed to explore the knowledge, attitudes and beliefs surrounding preconception health in men and women in the childbearing years, including those with and without children already. Findings will help contextualize public perceptions of preconception health, and may influence and inform the development of future PCC interventions.

## Methods

### Focus group setting and topics

A descriptive qualitative focus group methodology was chosen due to the exploratory nature of the research question [20, 21]. Full ethical approval was granted from the Queen’s University Belfast, School of Biological Sciences Research Ethics Committee. Five focus groups were recruited: two rural and three urban, conducted across Northern Ireland (NI), UK, to ensure a broad range of views (see Table 1 Focus Group Breakdown). Due to challenges recruiting participants, particularly males, or those without children, two focus groups were mini focus groups (i.e. three to four participants) or included both sexes. The inclusion criteria were: males or females of childbearing age (aged 18 to 45 years), drawn from an urban or rural area. Exclusion criteria were: participants suffering from chronic illness such as diabetes or polycystic ovarian syndrome (PCOS) which may affect pregnancy and/or childbirth, as these adults may be likely to receive additional advice regarding PCC and pregnancy planning. Groups were also organized to represent a range of views (see Table 1).

**Table 1:** Focus Group Breakdown

Focus Group Location	Sex	Age Range (years)	With/Without Children
Rural Group 1	Males and Females	18–45	With children
Urban Group 1	Males and Females	18–45	With and without children (mixed)
Urban Group 2	Females	18–45	Without children
Urban Group 3	Males	18–45	With and without children (mixed)
Rural Group 2	Males and Females	18–45	Without children

Participants were recruited using: (1) recruitment posters displayed in public areas in both urban and rural locations, and, (2) via email circulars to all levels of University staff and students. Interested participants contacted the project researcher to express their interest in taking part. They were sent an information sheet and contacted again in 48 h. If interested in participating, they were screened for suitability according to the inclusion and exclusion criteria and their availability to attend a focus group. Eligible participants were then invited to a specific focus group. Prior to beginning each focus group participants completed an informed consent process and a background demographics questionnaire. The questionnaire included information on age, sex, number of children, lifestyle and health. It assessed the extent of pregnancy planning in all participants who reported having children (males and females) using the London Measure of Unplanned Pregnancy (LMUP) [22]. This is a six-item measure with established psychometric properties that scores pregnancy planning or intention from 0 to 12. Scores of 0–3 were categorized ‘unplanned,’ 4–9 as ‘ambivalent’ and 10–12 as ‘planned’ [6, 22]. To assess dietary quality, a validated measurement tool was used; the Eating Choices Index (ECI) [23]. ECI includes four items with scores ranging from 0 to 20. A higher score indicates a higher quality of diet [23].

All five focus groups were conducted by two females (LM and ELC) and varied in terms of timing, e.g. afternoon or evening and location, University setting (urban location) or community hall (rural location). The lead facilitator (LM) was trained and experienced in qualitative methods. A semi-structured topic guide included: understanding/knowledge of preconception health, personal health status, learning about preconception health, seeking preconception help and learning about it in the future (see Table 2). Leaflets with information regarding further information and support on the topic of preconception health were given to all participants at the end of each focus group based on reputable websites such as the UK National Health Service (NHS) and leading charities in the field e.g. Tommy’s.

**Table 2:** Semi-structured topic guide for focus groups

	Questions
Understanding/ knowledge of preconception health	• Can you describe in your own words, what does the term “preconception or pre-pregnancy” mean to you?
	• What opportunities have you had where preconception care has been discussed, if any?
	• What factors would you consider to be important when planning for a baby, if any?
	• Have you done any research on how to improve your health to maximize the chances of having a healthy pregnancy?
	*For focus group where participants already had children:
	• Did you make any changes before trying? Are there any questions you would have liked to know more about but didn’t? Why?
	• What do you think contributes to positive preconception health?
Personal health status	• How would you rate yourself in terms of your overall health, considering diet, physical activity, alcohol intake and other lifestyle choices etc.?
	• How confident do you feel that you could make changes to improve your health before having a baby, if you choose to do so?
Learning about preconception health	• How do you believe you have learnt what you already know about preconception health? (Where, when, from whom?)
	• Have you heard about it from any other sources?
	• Do you feel that was the most effective way to learn or would you prefer to learn in a different way?
	• Do you feel that there are any life stages where it may be/have been beneficial to have a better understanding about preconception health? Via what mediums?
Seeking preconception help	• What would encourage you to seek help regarding preconception health, if anything? Has this changed over the years?
	• Why do you think so few people turn to a healthcare professional about the best things they can do for health only once they become pregnant, or are having trouble getting pregnant?
	• Where would you turn to if you wanted information on preconception health? What sources?
	• Have you thought about a baby being born with problems/health risks that could have been prevented via lifestyle changes? • Are there things/topics relating to preconception health that you would not want to discuss with a healthcare professional?
Future – improving knowledge	• Would anything motivate you to learn more about PCC? • If you decided to make health improvement before trying for a baby, how far in advance would you likely do this? Why?

### Data analysis

The focus groups were recorded and transcribed verbatim by a professional transcription company into Microsoft Word and verified by the research team (LM, ELC). A descriptive thematic approach was taken to the data analysis [24] which initially involved reading and re-reading of all transcripts. Coding was undertaken, with all transcripts being double coded by at least two researchers, and two of the five transcripts coded by all three researchers (LM, ELC, and CC). Codes were cross-checked regularly between researchers via a series of meetings. Codes were then examined and grouped into themes, with common themes between the focus groups identified using thematic analysis [24]. Codes, themes and sub-themes were compiled predominantly by ELC and discussed in detail with LM and CC regularly, and agreed in a process of triangulation (LM, ELC, and CC). The study was conducted and reported in line with the consolidated criteria for reporting qualitative research (COREQ) guidelines [25].

## Results

### Focus groups

Five focus groups were conducted across NI between October 2018 and July 2019, involving *N* = 21 participants; *n* = 8 males and *n* = 13 females aged between 18 and 45 years, with *n* = 8 (38%) participants reporting they had at least one child (see Table 3 for sociodemographic profile and health status of the sample). Each focus group lasted an average of one hour. The majority of participants were single females, without children, aged between 18 to 25 years.

**Table 3:** Sociodemographic characteristics and health behaviour status of focus group participants (*N* = 21)

Characteristic		n	%
Sex			
Male		8	38
Female		13	62
Age Category *(years)*			
18–25		11	52
26–35		4	19
36–45		6	29
Marital Status			
Married		4	19
Single		13	62
Divorced		1	5
Living with partner		3	14
Highest Education Level Achieved			
Secondary school (age 15/16 years – e.g. GCSE)		1	5
Secondary school (age 17/18 years – e.g. A-Level)		1	5
Additional Training (NVQ, BTEC, other)		3	14
University Undergraduate/Nursing Degree		9	43
University Postgraduate Degree		7	33
Occupational Status			
Full time paid work (30h hours per week)		8	38
Part time paid work (8–29 h per week)		3	14
Full time higher education		9	43
Unemployed		1	5
Pre-existing Medical Conditions			
Yes		2	10
No		19	90
Have Child/Children			
Yes		8	38
No		13	62
Health Behaviours and Pregnancy Planning Measure			
Self-described Weight Status			
Slightly underweight		2	10
About the right weight		13	62
Slightly overweight		6	19
Smoking Status			
Ex-smoker		2	10
Never smoked		19	90
Alcohol Intake			
Moderate drinker		10	48
Minimal drinker		10	48
Do not consume alcohol		1	4
	Mean (Median)	Scoring Range	
Eating choice index (ECI)^a^	14.39 (14)	11–18	
London Measure of Unplanned Pregnancy (LMUP)^$^	9.75 (10.5)	7–12	

^a^Measure of dietary quality: the Eating Choice Index (ECI) includes four items with scores ranging from 0 to 20. A higher score indicates a higher quality of diet [23]

^$^Measure of pregnancy planning for those with children already (*n*=8): London Measure of Unplanned Pregnancy (LMUP), a six-item measure with established psychometric properties that scores pregnancy planning or intention from 0 to 12. Scores of 0–3 were categorised ‘unplanned,’ 4–9 as ‘ambivalent’ and 10–12 as ‘planned’ [22]

Responses to the background questionnaire indicated 62% of participants identified as ‘about the correct weight’. No participants identified as smokers and the alcohol intake ranged from moderate drinker to not consuming alcohol. The mean and median scores for dietary quality as assessed by the ECI were 14.4 and 14.0 respectively, out of a possible 20 (range: 11–18), indicating relatively high dietary quality.

The mean and median values for LMUP in this sample (across *n* = 8 participants) were 9.75 and 10.5 respectively (range: 7–12) (see Table 3). This indicated that participants were on the borderline between ambivalent and planned, but with the majority (63%) of respondents scoring in the ‘planned’ category [22].

Five themes were identified from across the five focus groups, each of which are discussed in detail below.

### Theme 1: education surrounding preconception health

A small number of participants, mainly younger, discussed how they had encountered information on preconception health broadly or heard about PCC strategies (e.g. females taking folic acid before conception) in a secondary school setting (aged 11/12 years plus) or in third level (University) education, although the majority of participants in this research had not encountered the topic via education. In particular, preconception health was mentioned within a Biology or Home Economics context at more senior years in school (i.e., aged 15 years plus), and this would only be for the minority of students who selected these subject for study, not the whole student cohort.

*‘I did it in Biology A-Level and would have touched on it again during my university*

*course’ (Male respondent, urban group)*

*‘There was nothing really put out there in education for me [regarding preconception health]’ (Male respondent, rural group)*

A small number of participants said that due to the type of school they attended they were sheltered from this type of knowledge. Some stated a distinction between grammar and secondary schooling – where different subjects and topics were prioritized depending on the setting, for example:

*‘I went to a Convent, our education focused more on not getting pregnant!’*

*(Female respondent, urban group)*

*‘… it was in grammar school…we would have got nothing [on PCC] apart from some theory in biology and HE [home economics].’ (Female respondent, rural group).*

A minority of younger female participants (without children) also suggested that they didn’t feel the timing was right for them to be educated about preconception health or PCC strategies, and that this would have more resonance once a conscious decision to conceive had been made.

*‘I think it would motivate you [PCC] when you wanted to have a child, like I don’t really know if there would be any point in trying to educate me now on preconception health ‘cos I don’t think I would…actually really listen’.*

*(Female respondent, urban group)*

### Theme 2: Participant’s awareness of preconception health

Given the relatively limited exposure participants had regarding preconception health via formal education awareness was minimal, particularly among the male participants. Most males had never heard the term ‘preconception’ or given it any thought before attending the focus group. One respondent discussed how there is a lack of media attention on the topic of preconception health compared to the *‘incessant onslaught of media attention’ (Male respondent, urban group),* given to other health issues, such as smoking.

***‘****Never really thought about it [preconception health] before to be honest’.*

*(Male respondent, urban group)*

*‘I heard it [preconception health] about two weeks ago - whenever we were asked to take part [in this research]!’ (Male respondent, rural group).*

Overall, females appeared more aware of the terms preconception health or PCC and had a basic or superficial knowledge of certain aspects of important PCC behaviors. For example, requirements for folic acid consumption (for females) and following a healthy diet/good nutrition, though they were mostly unable to cite specific guidelines correctly.

*‘Nutrition [is important for preconception health] probably with the lifestyle choices that you make too, but I don’t really know [what else], probably more nutrition I would say’.*

*(Female respondent, rural group)*

*‘…we just hear [about] folic acid [but] I couldn’t name a specific rule or recommendation’ (Female respondent, urban group).*

There was also a lack of awareness regarding the male role in preconception health, with many believing mainly females contributed to good preconception health.

*‘I think it’s very much that the woman has to do all that because the woman’s carrying the baby,..‘(Male respondent, rural group).*

*‘I suppose equal opportunity…men should also take responsibility, but you’d never hear of that or anything’ (Female respondent, urban group).*

Awareness of preconception healthcare behaviors that might be beneficial in the period prior to conception were commonly confused with pregnancy. When asked about behaviors that may be important during the preconception period, a large number of participants recited pregnancy-related information e.g. risk of fetal alcohol syndrome, aside from knowledge about folic acid preconception.

*‘Folic acid and eating [certain foods] …If you do happen to get any illness throughout your pregnancy…and what you can and can’t do with certain food to minimize risks’.*

*(Female respondent, rural group)*

*‘Is it alcohol, fetal syndrome or something you call it, one of these can then cause the child to be... to have some deformities, but that’s more during pregnancy than before.’*

*(Male respondent, rural group)*

*‘Avoiding fish with high levels of mercury [preconception] which could affect the [baby’s] central nervous system’ (Male respondent, urban group).*

The majority of participants agreed that public awareness of preconception health and care needs to be improved within society, with some participants suggesting ways to increase public awareness of PCC, particularly with ideas to engage males in the PCC dialogue.

‘*I think making people aware of it [PCC] is definitely the first step’.*

*(Male respondent, urban group).*

Social media was discussed as a vehicle for passive knowledge improvement of preconception health, especially for males and those in the younger age groups, e.g., 18–25 years. For example, one participant stated if you saw a link or an interesting article title when browsing on a social media feed *‘you might just click on it’*:

*‘So, if it’s on Facebook, if there’s like an article or something that seems of interest…[you’re] going to look [at it]’.*

*(Male respondent, rural group)*

*‘I do read things like health things that come up anyway [on social media], but it’s only if they pop up, I mean I wouldn’t go for looking for it.’*

*(Female respondent, rural group)*

Quizzes via social media were also seen as something which may have appeal, e.g., a brief survey on your preconception health status with tailored feedback. Overall, it was felt that framing PCC health messages in a positive way was best to prompt behavior changes.

*‘I think a lot of people don’t like being told what to do nowadays, so instead of don’t be doing these things, it could be top 10 things to do to improve to your health. You know…ways to improve your health for wanting a child, if it’s more positive rather than negative it would definitely be better, I think.’*

*(Male respondent, rural group)*

*‘even a questionnaire that you could fill out and it scores your own health at the end to let you know where you are to see if you want to make changes [regarding PCC behaviors]’.*

*(Male respondent, rural group).*

### Theme 3: knowledge networks and sources of support regarding preconception health

#### Internet

In the minority of those who suggested they had already looked for some form of preconception health information (mainly those with children), there were a variety of methods employed. Mostly, this involved opting for individual/private research methods, i.e. by going online and using the internet, rather than approaching healthcare professionals in person.

*‘I did quite like chat rooms of people trying to get pregnant [when trying to conceive]… although it probably didn’t teach me much on preconception health really’ (Female respondent, urban group).*

Online/internet sources were commonly cited across all groups when asked where preconception health information could be found and included, ‘*Dr Google*’, the NHS website (UK) and Emma’s Diary (UK website), alongside chat rooms, forums and blogs from people using social media, e.g., Instagram and Facebook.

*‘I’d probably look at the internet first to get the general feeling on it [PCC]’.*

*(Male respondent, urban group)*

The majority of participants in this research identified that NHS approved sources were most reliable regarding information content, with chat rooms and forums being used more for entertainment purposes. Participants felt that social influencers/bloggers on sites such as Instagram had stories and opinions of interest, despite often not being medically qualified.

*‘[On Instagram influencers] though they’re not giving medical advice they still give good guidance sometimes, sort of hear other opinions and sort of [gives you] a rough idea.*

*(Female respondent, urban group)*

*‘[on Instagram] I think there is a blogger…the gynae-geek or whatever, and she…is all to do with [that]…a gynecologist is that what she is?’*

*(Female respondent, urban group)*

#### General practitioners (GPs)

A recurring point of view identified across focus groups was that seeing the GP (doctor) for PCC advice/strategies was somehow *‘wasting an appointment’,* and this, coupled with a lack of availability for GP appointments, put participants off:

*‘My concern would be that I would probably find myself thinking that it’s a less important use of GP’s time so maybe I should go find out as much information as possible myself before I use up their time…there are…other people who might need an appointment with health problems, rather than health improvement issues.’ (Female respondent, urban group).*

*‘I certainly wouldn’t be…going to a medical professional [preconception] unless you’re having problems [conceiving].’ (Female respondent, rural group).*

A minority of participants also felt that perhaps a GP wouldn’t have the specialist knowledge to help in detail with the issue of preconception health, leading others to suggest that specialist centers or clinics might be of more use.

*‘I probably would try and seek advice about it [preconception health] so you can have the most healthy pregnancy as possible, but I wouldn’t really know who to go to. I know you can go to the Doctor, but I don’t know if they would go in to the detail that maybe I’m looking for’.*

*(Female respondent, rural group)*

*‘maybe centres or whatever, that would help give information about having a child [would be useful]’.*

*(Male respondent, rural group).*

An emerging area discussed by a minority of participants (mainly younger males) was that of GP advice online, where you could essentially post anonymous questions to qualified health professionals without seeing them face-to-face.

*‘it can be anonymous [online], like you can speak to people anonymously about different things; doctors and stuff, get specialist information.’*

*(Male respondent, rural group).*

#### Family and social support

A high number of female participants suggested that they would consult family members and/or close friends (or had already done so), if they found themselves needing preconception health support or advice, whereas only a minority of males suggested this. There was a sense that if someone had already had a baby, they might have better advice on the topic, particularly if they struggled to conceive.

*‘I think mainly [I received PCC advice] from my mum because she had a lot of trouble with pregnancies and I think she maybe mentioned it more than other people might have.*

*(Female respondent, urban group)’.*

*‘Even like family or friends, that’s previously had a baby or something you could ask them [about PCC strategies] because they would know.’*

*(Male respondent, rural group)*

A difference in male and female perspectives on preconception advice/support was noted across the focus groups. No males suggested that they would approach male friends/family for preconception advice, perhaps due to a social stigma and/or pride issues, or that males may have a different relationship with their peers compared to females.

*‘I couldn’t imagine going to the boys at football and being like ‘aye [I’m] on the avocados now you know, I’m trying for a baby’… it’s not a topic of conversation that I’ve ever heard!’ (Male respondent, urban group).*

*‘Not ever [discussed preconception health] within my friend group, but I’ve seen on TV sometimes little bits where they say…for a man ‘trying’ [for a baby] then they should be trying to be healthy…’.*

*(Male respondent, urban group).*

### Theme 4: optimal parental health

Overall, the majority of participants in this sample felt that it was important to be in good health before having a baby. Most readily discussed common lifestyle factors which they felt had relevance to preconception health such as diet and nutrition, smoking, alcohol and stress.

*‘I suppose kind of having a healthy overall body I think would kind of be important I suppose trying to have a child, you’d want to be in your healthiest condition and for your child if you do become pregnant, would be…my idea of it anyway.’*

*(Female respondent, urban group)*

*‘To have a healthy pregnancy…to be able to get pregnant healthily and when you are pregnant…you shouldn’t be smoking or drinkin’ and all of that’.*

*(Male respondent, rural group)*

*‘[Stress] has a massive impact mentally and physically, so you’d have to try and minimise it where possible [preconception].’*

*(Male respondent, rural group)*

Participants identified dangers associated with smoking and alcohol, although again this was, at times, discussed in the context of ‘during pregnancy’. Participants displayed an awareness that alcohol should be minimized and smoking stopped when trying to conceive, for both males and females.

*‘I’ve seen smoking campaigns talk about how smoking affects male fertility and stuff- I think alcohol is the same…’ (Male respondent, urban group).*

*‘My sister’s husband was encouraged to stop smoking before they tried to have kids’ (Female respondent, urban group).*

*‘I’ve just heard about miscarriages and stuff with drinking alcohol and things like that and smoking causing...I don’t know if it’s a myth, about it [smoking] stunting growth and that sort of thing. I’ve heard that before.’*

*(Male respondent, rural group)*

However, many participants were aware that a lot of pregnancies are unplanned, so these steps aren’t always achieved preconception.

*‘…not everyone plans a pregnancy, some people it just happens, so they wouldn’t obviously have that time before to plan… they might not be thinking about it [changing lifestyle].’ (Female respondent, rural group).*

*‘If it [pregnancy] was planned, then before as well, yes, [stay away from drinking alcohol]… before and during [pregnancy]’.*

*(Male respondent, rural group)*

In addition, many participants discussed that good nutrition (including supplementation) and having a healthy weight or body mass index (BMI) was important for optimal preconception health, however, none were able to identify any specific reasoning behind this.

*‘Mostly it [PCC] relates to folic acid consumption… people who are considering it [pregnancy] might take some pre-natal vitamins or folic acid’.*

*(Female respondent, urban group).*

*‘But weight, fitness and nutrition are really the main things [for PCC], and stay away from bad habits.’*

*(Female respondent, rural group)*

*‘Well, I think weight management for both parties [males and females] would be paramount.’*

*(Male respondent, urban male)*

A minority of participants suggested that health professionals only provided specific PCC advice/strategies when an individual or a couple was having problems conceiving, or if a patient discussed actively considering getting pregnant.

*‘I went to the doctor to get the contraceptive implant out and my doctor was very straightforward [about PCC], they mentioned folic acid but said not to worry, these things [getting pregnant] take time’ (Female respondent, urban group).*

### Theme 5: attitudes and emotions surrounding preconception health

#### Timing of effort

A minority of participants (without children) exhibited an awareness of PCC strategies, e.g., moderating alcohol, not smoking and improving nutrition, but equally made clear that they would not consider incorporating these lifestyle changes at present due to timing, i.e., pregnancy was not ‘*on their radar’.*

‘*If you were actually trying to get pregnant maybe you would be a bit more [health] conscious’ (Female respondent, urban group).*

*‘If you were actively trying I think you would be more inclined to make the effort [to be healthy], but I think that at the minute it’s not in my radar’.*

*(Female respondent, urban group).*

#### Stress, perceived pressure and stigma regarding preconception health and subsequent pregnancy

The majority of respondents across groups perceived stress and pressure surrounding the issue of getting pregnant. Many stated they would not inform anyone (or had not previously) that they were trying to conceive. They felt this would reduce the pressure of being asked about how it’s going, especially in case it took longer than one might expect to conceive. A number of participants stated that this type of pressure was perhaps borne more by females, both in terms of personal health status, i.e., carrying the child, and also because there was a sense that females would discuss this type of issue more openly with one another, versus males.

‘*Yeah, aww there’s definitely more pressure on women’.*

*(Female respondent, urban group)*

*‘… I don’t think I ever told anybody beforehand [when trying to conceive], maybe because of the pressure idea, that it would put more stress on you, and I guess it’s quite private…’.*

*(Female respondent, urban group)’.*

There was a sense of fear that telling people when ‘trying’ would invite unsolicited opinions from others on how long it was taking as well as a fear that something may go wrong with the pregnancy in the early stages. Therefore waiting for the first scan (around 12 weeks) was preferred by some.

*‘I did [tell people early] with my first pregnancy and I regretted it then because it didn’t go the right way like, so I think yeah, I would wait [longer]’.*

*(Female respondent, rural group)*

*‘Yeah I think that’s the biggest thing, the fear like if you admit to trying [to conceive] and then you can’t, there’s a whole other repercussions of that kind of thing’.*

*(Female respondent, urban group).*

Male respondents across groups also displayed a reluctance to discuss preconception health or trying to conceive with their peers, for fear of straying into a female-dominated territory.

‘*I can imagine there would be a bit of a stigma around [discussing] pregnancy in general [with males] because it’s just perceived as a female-led operation’.*

*(Male respondent, urban group).*

Furthermore, when asked about the possibility of a baby being born with problems or health risks that may be attributable to parental lifestyle in the preconception period, there was a general sense of unease about this type of message framing. It was also felt that there was a disproportionate focus on the role women may play in this.

*‘They [people] would probably have blamed the woman if there were any problems [with a baby]’ (Male respondent, urban group).*

*‘It [PCC messaging] needs to be positive…you can’t say, you can’t be overweight or else your kids going to be overweight like, you need to be saying this is what could potentially happen but this is how and what you can do [about it]…it’s got to be really positive I think.’*

*(Female respondent, urban group).*

## Discussion

There is limited research qualitatively exploring the views and beliefs of adults of childbearing age on the topic of preconception health, particularly in the UK. This study explored the views of both males and females towards preconception health and PCC with thematic analysis across five focus groups identifying five themes: preconception education, preconception awareness, wider knowledge networks/support, optimal parental health, and attitudes/emotions surrounding preconception health. Key findings illustrated a lack of awareness of guidelines surrounding preconception health (for both sexes) and disparity between males and females regarding sources of support for preconception health. It highlighted that young adults do not feel comfortable consulting a doctor for general preconception advice, and illustrated a lack of awareness regarding the importance of male involvement in PCC behaviors.

It is concerning that amongst male participants, most were unaware of the biological importance of paternal health status in the preconception period. It was deemed a ‘*female led operation’*, yet research indicates that male fertility is closely linked to diet/nutrition and weight status [14]. This aligns with recent large-scale quantitative research from America which indicated that stereotypical beliefs were strong with regard to women upholding norms associated with PCC behaviors, and were significantly higher than beliefs about what men should do for PCC, among both female and male respondents [26]. Males were more likely to discuss online methods of information seeking, such as using social media quizzes and articles, or perhaps consulting online doctors for advice anonymously. This may indicate that concerns around stigma or pride pose a barrier for males to seek preconception health advice. Previous research suggests that men can be prompted to develop a ‘procreative consciousness’ and being able to visualize their future child and partner may help to influence their thinking prior to conception [27]. Ultimately, greater engagement in PCC behaviors for males may allow for optimization of biological, psychological and social factors. This has the potential to impact positively on men’s health and that off any potential offspring, enhancing the experience of fatherhood [28]. This is especially important, as research in the UK has shown that, in some instances, health behaviors are improved in the preconception period amongst males who report planning a pregnancy [29]. Yet the same study also reported that up to 57% of males took no action to improve their health before conceiving a baby which needs addressed [29]. The present research suggests that men indicate a willingness to become more involved in PCC behaviors and this should be capitalized upon. The best medium by which to do so however, is less clear. Results lean towards a preference for reputable online (anonymous) services and evidence-based social media spreading preconception health messages, rather than ‘influencer’ information from unqualified individuals.

The female participants from focus groups demonstrated some limited PCC knowledge on folic acid, with no participants able to identify specific requirements for the supplement. This is in line with quantitative evidence gathered across Europe, where a survey on women’s awareness and periconceptional use of folic acid indicated that 70% of over 20,000 female participants knew there were benefits of folic acid; only 17% however, could specify that folic acid reduced the risk of neural tube defects/spina bifida [30]. Getting into the *‘healthiest condition possible’*via diet/nutrition seemed a commonly shared view amongst participants, however, most were unsure of how nutritional needs differed between the preconception period and pregnancy itself. Many of those without children felt PCC lifestyle changes were something for *‘the future’*, given they were not at a life stage where they were considering pregnancy. This presents a challenge for preconception health promotion given the high proportion of unplanned pregnancies in females aged 20–34 years (62.4%) in the UK [16]. Previous research has shown that many women in the UK are not meeting the lower reference nutrient intake amounts for key vitamins and minerals, indicating that they will not be nutritionally prepared for pregnancy, should it occur [11]. Improving nutritional intake at a population level is needed alongside fostering an open culture of preconception healthcare in order to improve outcomes for unplanned and planned pregnancies. In England, calls have been made to initiate better annual reporting on preconception metrics using multiple sources of routinely collected data [31]. This could serve to hold governments and other relevant agencies to account for delivering PCC interventions to improve outcomes [31], as presently in the UK the services and resources needed have not yet been made available [31].

There was variability regarding participants’ views on an adequate timeframe to make positive changes to lifestyle and diet to be in ‘optimal body health’ for conception, often not aligned with research evidence. Participants’ suggestions ranged from a couple of months to a year for improved lifestyle before trying to conceive, yet studies have shown that dietary patterns up to three years before pregnancy can have an impact. For example, a high intake of fruit, vegetables, nuts, legumes and fish and a low intake of red and processed meats are associated with a reduced risk of hypertensive disorders of pregnancy, pre-term birth and gestational diabetes [11]. This further exemplifies the need for improved dietary intake at a population level alongside targeted individual-level interventions for preconception health. This aligns with current UK aims where a dual intervention strategy has been proposed to improve preconception health, targeting both the *public health level* (e.g., via improvements to the food environment) and also at the *individual level* (e.g., through better identification of those planning a pregnancy who would benefit from support to optimise health before conception) [31]. This would raise awareness of PCC behaviors and normalise planning and preparing for pregnancy [31]. In particular, the *individual level strategies* could reap significant gains for maternal and child health, given that the preconception period is often a time of high maternal motivation for behaviour change which may positively influence developmental (embryo) programming around the time of conception [31].

Enhanced ‘motivation’ for lifestyle improvement in the preconception period was evident across all focus groups; it was clear that key behaviors such as quitting smoking and possibly reducing alcohol intake were easily identified, although other lifestyle factors such as medication, weight management and nutrition were considered to play a role in preconception health. Understanding of exactly how, or why these behaviors would influence preconception health was limited across both males and females. Discrepancies arose regarding alcohol intake for males; some male respondents were of the impression that it was irrelevant whether or not they moderated alcohol intake in the preconception period, though appeared to place importance on quitting smoking. Preconception advice from the British Nutrition Foundation suggests that excessive alcohol intake may affect sperm quality, therefore men should follow the Department of Health’s (England) recommendation that males moderate their intake to a maximum of three to four units daily [32]. Regarding smoking, the message is clearer: males who smoke are likely to have reduced semen quality; they are advised to stop smoking if trying to conceive [32]. Perhaps the public perceive greater ambiguity around the importance of a behavior if asked to moderate or reduce it, rather than stop completely in the preconception period. Understanding the complexities of this would require further research.

Many participants across groups discussed the importance of a healthy weight for both males and females before trying for a baby, despite being unable to cite any direct impact of weight status upon pregnancy or conception-related outcomes. Clearly, public understanding of the importance of a healthy weight preconception needs improved, as research shows the risk of infertility is up to three times higher in women with obesity when compared to those without obesity, with the probability of pregnancy being reduced by 5% per unit of BMI exceeding 29 kg/ m^2^ [33]. Mechanisms of action which may be responsible for the negative impact of excess weight in the preconception period have included impaired ovarian follicular development, qualitative and quantitative development of the oocyte, fertilization, embryo development and implantation [33, 34]. Furthermore, evidence indicates that maternal *and* paternal obesity can predispose the unborn baby to diseases in adulthood and greater obesity risk as a result of fetal programming [35, 36]; this is an important shared responsibility public health message.

This research appears to support the need to promote preconception health to all males and females and participants suggested a number of strategies beginning with school-based education (e.g. within sexual and reproductive health aspects of the curriculum, as well as more broadly e.g. within biology, or home economics) and continuing throughout the life course. It supported having a particular emphasis on identifying and accessing those with an intention to conceive in the short to medium term future, in order to positively influence PCC behaviors [11]. A societal shift is required regarding an emphasis on preconception health in order to improve and extend men *and* women’s awareness to seek health professional advice at the preconception stage. Perhaps this would be facilitated by improved healthcare provision for this type of service, rather than adults feeling they are *‘wasting’* the GP’s time. Presently in the UK, GPs are advised to discuss preconception health with those who are planning a pregnancy (and their partners), yet this research suggests that this opportunity is being missed. Perhaps a broader health promotion approach to consider would be that of a Reproductive Life Plan, which has been implemented in America and Sweden with some positive findings [37].

### Strengths and limitations

Strengths of this study are that, to our knowledge, it is one of the first carried out within the United Kingdom focusing on qualitatively studying the views of both males and females of childbearing age (with and without children) with regards to preconception health. It provides in-depth, rich information on the issue which can be missed in quantitative methodologies. The background questionnaire used in the research further strengthens the ability to contextualize the findings by profiling the participant’s sociodemographic and health backgrounds. It indicated the sample had relatively high self-reported dietary quality, none were smokers, and all were moderate to non-alcohol consumers, which may limit generalizability to other groups. Some limitations of this study also relate to the sample size (*N* = 21); this was in part due to difficulties recruiting participants for focus groups, especially male-only groups and those without children. The framing of this type of research and where participants are sought should therefore be considered in future studies to negate this.

### Future research

Given some of the differences in opinion discussed here i.e., between males and females, and between those with and without children currently, it would be of interest to conduct further in-depth interviews on the topic, where people may feel they can more freely discuss contentious issues relating to preconception health. This should include adults drawn from across the socio-economic spectrum and with varying education levels. It would also be of interest to conduct research with healthcare professionals in NI, such as GPs, nurses and pharmacists on what type of advice they routinely offer on PCC behaviors, and how they feel about future dedicated PCC services for those planning a pregnancy. Furthermore, given the relatively small sample size, it was not possible to ascertain urban/rural differences of opinion, or indeed explore views in-depth based on child status, and whether views differed in those with children who had planned their pregnancies. These areas should be explored in future research.

## Conclusion

This study highlights the need to improve awareness of preconception health amongst males and females of childbearing age in the UK. Participants, particularly younger adults in this sample expressed a desire to learn more about preconception health and PCC strategies, perhaps indicating an opportunity to increase preconception health promotion and awareness. Suggested ways to improve awareness, particularly amongst younger participants, were using social media platforms, with positive framing of PCC messaging rather than fear or threat-based campaigns. The research also indicated a need to consider the most appropriate preconception educational strategies and the timing of these based on life-stages, in order to facilitate the development of a culture which values preparation for pregnancy, amongst males and females.

## Data Availability

The transcripts generated during and/or analyzed during the current study are available from the corresponding author upon reasonable request.

## Abbreviations

BMI: Body Mass Index
ECI: Eating Choices index
GP: General Practitioners
LMUP: London Measure of Unplanned Pregnancy
NHS: National Health Service (United Kingdom)
PCC: Preconception Care
PCOS: Polycystic Ovarian Syndrome
